# Contributions of Magnetoencephalography to Understanding Mechanisms of Generalized Epilepsies: Blurring the Boundary Between Focal and Generalized Epilepsies?

**DOI:** 10.3389/fneur.2022.831546

**Published:** 2022-04-27

**Authors:** Thandar Aung, Jeffrey R. Tenney, Anto I. Bagić

**Affiliations:** ^1^Department of Neurology, University of Pittsburgh Comprehensive Epilepsy Center (UPCEC), University of Pittsburgh Medical Center (UPMC), Pittsburgh, PA, United States; ^2^Division of Neurology, Department of Pediatrics, Cincinnati Children's Hospital Medical Center, Cincinnati, OH, United States

**Keywords:** magnetoencephalography, source localization, generalized genetic epilepsy, absence epilepsy, myoclonus epilepsy, epilepsy classification

## Abstract

According to the latest operational 2017 ILAE classification of epileptic seizures, the generalized epileptic seizure is still conceptualized as “originating at some point within and rapidly engaging, bilaterally distributed networks.” In contrast, the focal epileptic seizure is defined as “*originating within networks limited to one hemisphere*.” Hence, one of the main concepts of “generalized” and “focal” epilepsy comes from EEG descriptions before the era of source localization, and a presumed simultaneous bilateral onset and bi-synchrony of epileptiform discharges remains a hallmark for generalized seizures. Current literature on the pathophysiology of generalized epilepsy supports the concept of a cortical epileptogenic focus triggering rapidly generalized epileptic discharges involving intact corticothalamic and corticocortical networks, known as the *cortical focus theory*. Likewise, focal epilepsy with rich connectivity can give rise to generalized spike and wave discharges resulting from widespread bilateral synchronization. Therefore, making this key distinction between generalized and focal epilepsy may be challenging in some cases, and for the first time, a combined generalized and focal epilepsy is categorized in the 2017 ILAE classification. Nevertheless, treatment options, such as the choice of antiseizure medications or surgical treatment, are the reason behind the importance of accurate epilepsy classification. Over the past several decades, plentiful scientific research on the pathophysiology of generalized epilepsy has been conducted using non–invasive neuroimaging and postprocessing of the electromagnetic neural signal by measuring the spatiotemporal and interhemispheric latency of bi-synchronous or generalized epileptiform discharges as well as network analysis to identify diagnostic and prognostic biomarkers for accurate diagnosis of the two major types of epilepsy. Among all the advanced techniques, magnetoencephalography (MEG) and multiple other methods provide excellent temporal and spatial resolution, inherently suited to analyzing and visualizing the propagation of generalized EEG activities. This article aims to provide a comprehensive literature review of recent innovations in MEG methodology using source localization and network analysis techniques that contributed to the literature of idiopathic generalized epilepsy in terms of pathophysiology and clinical prognosis, thus further blurring the boundary between focal and generalized epilepsy.

## Introduction

Epilepsy is one of the most common neurological disorders affecting almost 3.5 million in the USA and 65 million worldwide and is getting increased public health attention as patients with epilepsy have a noticeable reduction in quality of life and employment prospects (1). Two major classification categories are whether an epilepsy is focal or generalized. According to the latest operational 2017 ILAE classification of epileptic seizures, the generalized seizure is still conceptualized as “*originating at some point within and rapidly engaging, bilaterally distributed networks.”* In contrast, the focal seizure is defined as “*originating within networks limited to one hemisphere*” (2). The definitions for generalized and focal seizures are retained from the 1981 ILAE classification, and the presumed simultaneous bilateral onset and bisynchrony of the epileptic discharges in electroencephalography (EEG) remains a hallmark for generalized seizures (2–4).

In 1952, Tükel and Jasper et al. reported that a mesial frontal cortical lesion could give rise to diffuse interictal spike-and-wave discharges; hence the term “secondary bilateral synchronization” and blurring the boundary of focal and generalized epilepsy (5). In addition to the frontal lobe, focal epilepsy with rich connectivity, such as posterior parietal, temporal, or even occipital lobe epilepsy, can give rise to diffuse “generalized” spike and wave discharges (GSWD) resulting from widespread bilateral synchronization, especially in the pediatric population and can be misclassified as generalized epilepsy (6–10). On the contrary, current literature validates that focal EEG features can be found in generalized epilepsy (11). Consequently, generalized epilepsy can also be misclassified as focal epilepsy. Therefore, making a distinction between generalized and focal epilepsy may be challenging in selected clinical cases (7–10). However, the 2017 ILAE classification proposes the combined generalized and focal epilepsy as one of the categories of the epilepsy classification for ambiguous cases. Nevertheless, treatment options, such as the choice of antiseizure medications (ASMs), neuromodulation, or surgical treatment alternatives, are the reason behind the importance of accurate differentiation between focal and generalized epilepsy. When epilepsy becomes drug-resistant, defined as failure to control the seizures with two appropriate ASMs, surgical resection or disruption of the epileptogenic zone (EZ) may be a way to achieve seizure freedom or reduce seizure burden in focal epilepsy, but those with generalized epilepsy are often not considered as epilepsy surgery candidates (12–16). Although neuromodulatory treatments such as vagal nerve stimulators (VNS) (17), responsive neurostimulator (RNS) (18), and deep brain stimulators (DBS) (19) are treatment alternatives for those patients who are not resective surgical candidates, the study and indications of all those neurostimulators are mostly based on focal epilepsy (20). Thus, the treatment that we could offer for drug-resistant generalized epilepsy is more limited than for focal epilepsy. There have been multiple works of literature supporting the usage of the neurostimulators, especially the RNS and DBS targeting different parts of the thalamus and cortices in patients with generalized epilepsy. However, the outcome is highly dependent upon electrode placement in relation to different thalamic nuclei, stimulation parameters, subtypes of generalized epilepsy, or even individual cortical-subcortical connectivity profiles (20, 21).

To improve the treatment options in generalized epilepsy, plentiful scientific research on its pathophysiology has been conducted over the past several decades, using advanced non–invasive investigations and postprocessing of the neuromagnetic signal by reflecting the spatiotemporal and interhemispheric latency of bi-synchronous or generalized epileptiform discharges in both animals and humans (22–24). Using invasive intracranial electroencephalographic (icEEG) data, Chen et al. reported that two hemispheres could still function independently with different focal network structures and properties under a strong global epileptic network in generalized epilepsy; the focal epileptic network from the leading hemisphere might be activating the global epileptic network. By resecting the part of the region of the leading hemisphere, five pediatric patients with generalized epilepsy with tonic/atonic and atypical absence seizures resulted in seizure freedom (24). Although the diagnosis of generalized epilepsy in the case series was disputable as all the resective brain tissue showed abnormal pathology (3 with focal cortical dysplasia type 1A, one with focal cortical dysplasia type 1B with polymicrogyria, and one with tuberous sclerosis), the conclusion was based on EEG and clinical semiology of the seizures. non-etheless, the author highlighted one of the current clinical challenges in accurately categorizing the epileptic patients into either focal or generalized epilepsy (24). In addition, accumulating evidence has shown that epilepsy is an archetypical neural network disorder. With ongoing debates, current literature on the pathophysiology of generalized epilepsy supports the concept of a cortical epileptogenic focus triggering the rapidly generalized epileptic discharges involving intact corticothalamic and corticocortical networks, which is known as the *cortical focus theory* (22, 23, 25).

Among all advanced non–invasive techniques, magnetoencephalography (MEG) provides excellent temporal and spatial resolution, inherently suited for analyzing the propagation of generalized EEG activities and determining whole-brain functional connectivity network patterns (26–29). The current clinical application of MEG for epilepsy in the form of magnetic source imaging (MSI) mostly uses the single equivalent current dipole (ECD) model (30, 31), especially in the United States (32). However, the traditional ECD model is restricted if the underlying assumption of focality is not fulfilled, for example, when the epileptiform activity occurs simultaneously across the various regions (33–35). Alternative source localization techniques, such as beamformer and low-resolution brain electromagnetic topography (LORETA), as well as various connectivity analyses, have played a prominent role in improving the localization of deep sources (further details in section 2) (34–40). Although there is an overall improvement in the strength of localization of the neuromagnetic activity using various source localization algorithms, there are still major limitations in analyzing deep sources with MEG (41–44). The magnetic field intensity is inversely proportional to the square of the distance between the sources and the sensors (45), and thus there is decreased signal in deeper structures of the brain, either deep cortices or the thalamus. Since GSWDs typically have very high voltages, it is postulated that MEG may be able to overcome this particular limitation in generalized epilepsy. Unfortunately, there are limitations in precise localization of the deeper structures, such as individual thalamic nuclei. Compared to MEG, functional magnetic resonance imaging (fMRI) has a better spatial but weaker temporal resolution (46). With advances in technology, there have been publications (40, 44, 47, 48) focusing on the multimodal integration of MEG with other neuroimaging techniques, mainly fMRI, to complement one modality with the other to further edify the underlying pathophysiology of the GSWDs.

In this review article, we searched PubMed, Medline, and Embase databases using the following search algorithm: “Magnetoencephalography (MEG)” and “Generalized Epilepsy” or “absence epilepsy” or “myoclonus epilepsy” or “generalized genetic epilepsy” limited to publications in English. The last date of the search was September 30th, 2021. We screened the titles, abstracts, and references of all search results to identify potentially relevant studies. We included only publications of MEG recordings in human subjects. We excluded poster publications and any study with abnormal MRI in generalized epilepsy patients. This article aimed to provide a comprehensive literature review of how the recent innovation in the MEG methodology contributed to the literature of the idiopathic generalized epilepsy in terms of physiopathology, treatment, and prognosis, thus further blurring the boundary between focal and generalized epilepsy.

## Different Source Localization Models, Techniques and Connectivity Analyses

Cohen et al. were the first to record neural magnetic signals using a single-channel MEG (49). Since then, the MEG recording technique has been enhanced, and now the neural magnetic signals can be recorded using more than 200–300 sensors (31, 43). With this advancement, source estimation models have been developed to localize the neural magnetic signals (26, 30–32, 37, 39, 40, 43, 50). Source analysis usually occurs in the source space rather than sensor space, where the neural signal is acquired at each measurement sensor. Due to the various ambiguities associated with sensor level analysis, source analysis is preferred, but sensor level analysis can be performed when there are not enough sensors, e.g., analyzing 10–20 EEG neural signals, to accurately localize sources (42, 51). The goal of source localization is to correctly estimate the location and orientation of the neuromagnetic source using the inverse model (37). Multiple mathematical algorithms have been developed, but non-e is felt to be superior for every clinical situation. Each algorithm comes with its own advantages and limitations. Besides, there have been studies showing overall agreement in estimating the sources when compared to intracranial EEG (52–54).

### Dipole Model

Dipole models are characterized by a single or few neural sources that are analyzed in the brain model and then sequentially moved until the projected single pattern matches the recorded pattern (30, 31). Among all, the single equivalent current dipole model (sECD) is the most well accepted, but the traditional ECD model is limited if the underlying assumption of focality is not fulfilled (30–32). Using point source analysis, the dipole modeling becomes limited and unreliable if the source is complex, multiple sources are generated over the same temporal course, or the source is generated from extended areas of the brain (26, 33–35, 55).

### Multiple Signal Classification

Multiple signal classification (MUSIC) can analyze complex and asynchronous sources that typically require multiple simultaneous source localization by scanning all possible positions of the brain in three-dimensions. However, two assumptions need to be met for accurate localization, an absence of noise and an accurate head model (34, 56, 57). Unlike ECD, the recursive MUSIC (R-MUSIC) algorithm can localize multiple synchronous sources using the spatio-temporal independent topographies (IT) model (58).

### Beamforming

Instead of estimating or reconstructing the source distribution, beamforming uses spatial filters to optimize predefined regions of interest or sources with a maximum signal while suppressing activity from other regions, including noise (59). Beamforming can be further divided into either linear, linearly constrained minimum variance (LCMV) (60) or non-linear, synthetic aperture magnetometry (SAM) (61). LCMV beamforming can be analyzed either in time domain using covariance metrics or in the frequency domain using cross-spectral density metrics, such as dynamic imaging of coherent sources (DICS) or accumulated spectrograms, such as accumulated source imaging (ASI) (59–63). Compared with ECD, beamforming can analyze multiple sources, either synchronous or asynchronous. Contrary to MUSIC, the neural signal analyzed with beamforming is less altered by the presence of noise. One of the limitations of beamforming, especially SAM with excess kurtosis, is performance loss when the sources are correlated. In addition, SAM with excess kurtosis [SAM_(g2)_] ignores frequent events, and thus it is limited in analyzing frequent discharges (59, 61).

### Current Density Models

Current density models directly compute a current distribution throughout the full brain volume: minimum norm estimate (MNE), standardized low-resolution brain electromagnetic tomography (sLORETA), exact low-resolution brain electromagnetic tomography (eLORETA), sLORETA weighted accurate minimum norm (SWARM), dynamical statistical parametric mapping (dSPM), and the multiresolution focal underdetermined system solution (MR-FOCUSS) (64–67). In MNE, dipoles are analyzed simultaneously in two-dimensions by limiting the space so their strengths can be estimated as the function of time (68). MNE has an excellent spatial resolution for the superficial sources, especially complex sources, but not for deeper sources due to the model limitation (69). To improve the superficial source bias, sLORETA performs further post-processing of the current density map obtained from the MNE by replacing the noise covariance with theoretical data covariance (65). Another normalization method of the MNE current density is dSPM which computes the normalization based on the noise covariance (70, 71). In addition, the presence of biological noise has no localization bias in the source estimation of the neural signals by sLORETA (67). dSPM and LORETA improve the localization error when compared to MNE (67, 72). To improve the analysis of complex dynamic sources using the time domain, particular models are a promising technique for the ictal dynamic data, especially MR-FOCUSS (73).

### Entropy Measures

Maximum entropy on the mean (MEM) is a technique to analyze synchronous sources in specific frequency bands and is sensitive to spatially extended sources using data-driven parcellation of the cortical surface into non–overlapping parcels. By maximizing the entropy of a probability distribution, the parcels that are not contributing to the measured data are excluded from the analysis (74). Pairwise MEM (pMEM), a statistical model of pairwise regional coactivation from empirical data using frequency-specific MEG resting oscillatory activity, can analyze the dynamic state's multi-stability (75, 76). The limitation of MEM is that a priori information on the number of cortical parcels is required (77).

### Connectivity Measures

Over the past decades, studies have been focused on analyzing various cortical networks using diverse connectivity measures to describe the disruption of the disease state from the normal functional neural networks (78–80). In contrast to anatomical connectivity, where networks of physical white matter structural connections or synaptic connections between various (distinct) regions of the brain analyzed at the micro or macroscopic occurs, functional and effective connectivity describes the functional aspects of neural networks (81). Functional connectivity measures the temporal correlation of distinct cortical regions, whereas effective connectivity analyzes the direction of the influence of one cortical region over distinct cortex (79, 81–86). Therefore, functional connectivity analyzes whether neural activities of the two regions are linked, i.e., undirected information flow, while effective connectivity scrutinizes the direction of the communication, i.e., directed connectivity (87–89).

Correlation and coherence are the most classical measures of functional connectivity and analyze the similarity between neural signals in the time and frequency domains, respectively (90). Other functional connectivity methods are based upon quantifying the waveforms in amplitude and oscillations of neural activity, such as phase lag index, phase slope index, or phase-locking index (91, 92). If the directional interactions are pre-defined, structural equation modeling (SEM) can be used, whereas Granger causality measures the connectivity on directional interactions derived from the data (82, 89, 93). Other effective connectivity methods are directed coherence, dynamic causal modeling, linear non–Gaussian, conditional Bayes, and Bayes network methods (94–99). The main difference between functional and effective connectivity is that functional connectivity illustrates statistical dependencies, whereas effective connectivity is based on a mechanistic model of the causal effects that generated the data (87, 100).

Graph theory provides models of complex dynamic networks in the brain, allowing one to better understand the relations between and/or the processes taking place in network structures. After the connectivity matrices are calculated, these values can be used to describe features of the network using graph theory, i.e., the network is defined as a set of nodes that are connected by edges or lines. This allows the investigator to calculate measures of different graph metrics, such as degree (number of connections to a node), node strength, path length, global efficiency, clustering coefficients, between centrality, synchronizability, small world index and centrality, to identify the critical components of the network (101–105).

## First Clinical MEG Recording in Generalized Epilepsy (Source Localization ERA)

Hughes et al. were the first to report a clinical MEG recording of 3 Hz generalized spike and wave discharges (GSWDs) in humans using simultaneous EEG and MEG recording (106). Interestingly, they observed that MEG was excellent in displaying the spikes and less evidence of waves when compared to the EEG. In addition, MEG waveforms were noted to precede the corresponding EEG spike activity in most patients' recordings. Ricci et al. studied the 3 Hz spike-wave using single-channel MEG with a phantom brain model and showed cortical activity was scattered bilaterally, mainly over frontal and temporal regions, often with more involvement over one hemisphere, while bilateral synchronous activity seemed to have originated from a deeper structure (107, 108). The study was the first to demonstrate evidence of primary cortical involvement in GSWDs in generalized epilepsy using neuromagnetic cortical source localization. The authors couldn't explain the relation of the cortical source localization to the deep brain structures given the limitation of the applied methodology with single-channel recordings (109). Thus, the author recommended further studies using multichannel capability with newer post-processing methodology to glean greater insights into the pathophysiology of generalized epilepsy (109).

## Childhood Absence Epilepsy

Childhood absence epilepsy (CAE) is the most studied generalized epilepsy among all genetic or idiopathic generalized epilepsy subtypes. All the published study characteristics, types of post-processing signal analysis, and main results are summarized in Table 1.

**Table 1:** Showing all the published study characteristics and main outcomes on childhood absence epilespy.

**Article name**	**Type of genetic epilepsy**	**No. of patients included in study**	**No. of female (F): No. of male (M)**	**Study State of genetic epilepsy**	**Mean age at the time of MEG recording (range) (y)**	**Mean age of epilepsy onset (range) (y)**	**Duration of epilepsy (range) (m)**	**No. of pt. on ASM**	**Yes and No for the Simultaneous MEG/EEG recording** **No. of EEG, MEG sensor with sampling rate of the MEG recording** **Source or sensory level (for the connectivity study only)**	**Type of analysis with analyzed MEG frequency bandwith**	**Main result**
**CAE (interictal/ictal GSWDs)**											
Westmijse et al. (110)	CAE	5	2F: 3M	Ictal	9.5 (7–12)	NA	NA	5	Yes (EEG−28, MEG 151 for the first 4 patients and 275 for the 1 patient) (1,200 Hz)	Beamformer (SAM) (1–70 Hz)	• Beamformer technique supported the local or even focal cortical involvement in the occurrence of the spike in the train of GSWDs. •GSWDs had local frontal and parietal cortical sites before the onset of the generalized pattern of GSWDs
Hu et al. (111)*	CAE	13	10F: 3M	Ictal	8.4 (0.17-−12)	NA	1.6 (3–36)	NA*	No (MEG 275) (1,200 Hz)	Beamformer (SAM) (20–70 Hz)	• Cortical epileptic foci were localized only 5 out of 13 cases over the bilateral frontal regions.
Tenney et al. (112)**	CAE	12	7F: 5M	Ictal	8.8 (6.4–11.8)	8.8 (6.4–11.8)	~ one week	0	Yes (EEG– 25, MEG −275) (4,000 Hz)	Beamformer (SAM), sLORETA (1–70 Hz)	• Beamformer analysis using SAM confirmed the presence of the independent thalamic and cortical activities. •sLORETA analysis showed sources during the absence seizures are most likely to be localized to the frontal cortex and thalamus at −50 ms.
											• At the onset of the absence seizure (0 ms), focal source localization was seen in the lateral frontal cortex with decreased thalamus localization.•Following the onset of the spike, localization between more widespread and gradually recruited throughout the cortex.
Tenney et al. (113)^**^, ^#^	CAE	12	7F: 5M	Ictal	8.8 (6.4–11.8)	8.8 (6.4–11.8)	~ one week	0	Yes (EEG– 25, MEG −275) (4,000 Hz)	Time-frequency analysis with different frequency bandwidths (1–20, 20–70, 70–150 Hz), sLORETA	• First to report on the frequency-dependent nature of the neural network and about HFO •During the absence seizure, frontal cortex source localization was noted at the low– (3–20 Hz) and gamma-frequency bandwidths (70–150 Hz). •At low-frequency bandwidths, more sources localized to the parietal lobes during absence seizure.
Jacobs-Brichford (114)^#^	CAE	12	7F: 5M	Preictal	8.8 (6.4–11.8)	8.8 (6.4–11.8)	~one week	0	Yes (EEG−23, MEG−275) (4,000 Hz)	sLORETA (1–70 Hz)	• Preictal MEG frequency changes were detected at a mean of 694 ms before the initial GSWDs on the EEG, and focal sources were localized to the frontal cortex and thalamus
Miao et al. (115)	CAE	14	9F:5M	Ictal	8.5 (5–12)	NA	7.1 (1–24)	0	No (MEG−275) (300 Hz)	Beamformer (wavelength-based), Dynamic magnetic source imaging (dMSI) (1–140 Hz)	• Initial ictal activity was source localized predominately to left frontal and posterior cortices. Frontal sources were left medial prefrontal cortex, pre-SMA, primary motor cortex, and lateral prefrontal cortex. The posterior cortical regions were the left precuneus and medial occipital cortex.
											• After initialization, the ictal activity showed involvement of medial prefrontal cortex and precuneus, and recursive propagation between frontal and posterior cortices *via* either medial portion of the brain (9/14) or thalamus (5/14), respectively.
Miao et al. (116)	CAE	10	7F: 3M	ictal	8.3 (5–11)	NA	5.9 (1–12)	0	No (MEG−275) (6,000 Hz)	Beamformer (wavelength-based), Dynamic magnetic source imaging (dMSI) (14–70, 80–500 Hz)	• HFO ranging from 80–500 Hz was located in all patients. •The total time of fast ripples (250–500 Hz) was greater than that of ripple (80–250 Hz) during absence seizures. •Compared to spikes, the source localization of HFOs appeared to be more focal.
											• HFO duration was significantly longer when co-occurring with spikes and localized in the medial prefrontal cortex, whereas spikes were widespread to the various brain regions during the seizure. •HFO (fast ripples) was associated with increased seizure frequency
Xiang et al. (117)	CAE	10	3F: 7M	Interictal	8 (6.4–10)	8 (6.4–10)	~one week	0	No (MEG−275) (4,000 Hz)	Beamformer (ASI), correlation analysis at Source level with multi-frequency analysis (1–4, 4–8, 8–12, 12–30, 30–55, 65–90, 90–200, 200–1,000, 1,000–2,000 Hz)	• Compared with healthy control, CAE patients had higher odds of interictal HFO in 200–1,000 and 1,000–2,000 Hz in medial frontal regions and parieto-occipito-temporal junction.
Tang et al. (118)	CAE	12	8F: 4M	Preictal/ ictal	8.17 (5–12)	7.75 (5–11)	7.08 (1–20)	0	No (MEG−275) (6,000 Hz)	Beamformer (ASI), correlation analysis at Sensor and source level with multi-frequency analysis (1–4, 4–8, 8–12, 12–30, 30–45, 55–90, 90–200, 200–1,000 Hz)	• Interictal to ictal period, neuromagnetic changes predominantly occurred in the medial prefrontal cortex and parieto-occipito-temporal junction at the low-frequency band at <30 Hz. •A strong correlation between the source strength of ictal HFOs in 200–1,000 Hz and the frequency of daily seizures was reported.
**CAE (Ictal network connectivity)**											
Gupta et al. (119)	CAE	5	NA	Preictal	9.5 (7–12)	NA	NA	5	No (MEG −151 for 4 patients, MEG −275 for 1 ptaient) (1,200 Hz) Connectivity– Source level	Beamformer (DICS), Graphic theory, non-linear coherence, source analysis (0–50 Hz)	• Beamforming showed a consistent appearance of a low-frequency frontal cortical source preceded by the low-frequency occipital source before the first ictal GSWDs.
										with low-frequency band 2–4 Hz and high-frequency band 20–25Hz	• There was a decrease in local connectivity and higher global connections at the preictal stage (1 s from the first ictal GSWD), suggesting a pathological predisposed preictal state toward synchronous seizures networks.
Wu et al. (120)	CAE	14	9F: 5M	Preictal	8.1 (5.3–11)	NA	8 (0.5–36)	0	No (MEG −275) (6,000 Hz) Connectivity– Source level	Beamformer (ASI), Graph theory, Granger causality, correlation analysis at source level with multi-frequency analysis (1–4, 4–8, 8–12,12–30, 30–80, 80–250, 250–500 Hz)	• At the preictal period, low frequency 1–80 Hz activities were localized to the frontal cortex and parieto-occipito-temporal junction, whereas high-frequency 80–250 Hz oscillations showed predominant activities localized in the deep brain region as well as medial frontal cortex. •Increased cortico-thalamic effective connectivity was observed around seizures in both low and high-frequency ranges.
											• At the early preictal period, the predominant direction of the cortico-thalamic effective connectivity was observed from cortex to thalamus, but the cortex that drove connectivity varied among subjects.
Youssofzadeh et al. (121)	CAE	16	9F: 7M	Preictal	8.7 (6–12)	NA	NA	0	Yes (EEG−25, MEG−275) (4,000 Hz) Connectivity-Sensor level	Beamformer (LCMV), Graphic theory, phase-locking value (PLV) at broadband frequency (1–40 Hz)	• During absence seizures, highly connected brain areas or hubs were present in the bilateral precuneus, posterior cingulate, thalamus, and cerebellar regions
Jiang et al. (122)	CAE	15	11F:4M	Ictal (termination)	(5–11)	NA	18.1 (2–63)	0	No (MEG−275) (6,000 Hz) Connectivity-Source level	Beamformer (ASI), Graph theory, Granger causality, correlation analysis at the source level	• At the seizure termination transition, activities at low frequency (1–80 Hz) were predominantly distributed in the frontal cortical and parieto–occipito–temporal junction, whereas high frequency
										with multi–frequency analysis (1–4, 4–8, 8–12, 12–30, 30–80, 80–250, 250–500 Hz)	(80–500 Hz) activities were localized in the medial frontal cortex and deep brain areas (mainly thalamus). •Cortico–thalamic effective connectivity was enhanced at all frequency bands, the direction of which was primarily from various cortical regions to the thalamus
Sun et al. (123)^θ^	CAE	22	15F: 7M	Preictal	8.5 (5–14)	NA	7.61 (4–13)	7	Yes (EEG−23, EEG−275) (6,000 Hz) Connectivity–Source level	Beamformer (ASI), correlation analysis at source level in 6 frequency bandwidths (1–4, 4–8, 8–12, 12–30, 30–80, 80–250 Hz)	• At the preictal stage (1 second from the first ictal GSWD), overall network spectral power increased and distributed at 2–4, and ictal spikes simultaneously showed elevation of network connectivity, predominately excitatory. •HFO was detected in certain focal areas
Sun et al. (124)	CAE	18	13F: 5M	Ictal (termination)	8.4 (5–11)	NA	10.2 (3–32)	0	No (MEG−275) (6,000 Hz) Connectivity–Source level	Beamformer (ASI), source–level with multi–frequency analysis (1–4, 4–8, 8–12, 12–30, 30–80, 80–250, 250–500 Hz)	• At seizure termination, low–frequency bands at 1–4, 4–8 and 8–12 Hz activities were distributed mainly in the frontal and parieto–occipito–temporal junction. At 12–30 and 30–80, there was significant reduction in source activity in frontal lobe. •The ictal peak source strength in 1–4 Hz was negatively correlated with seizure duration, whereas the 30–80 Hz range was positively correlated with epilepsy course
Tenney et al. (48)^#^	CAE	13	7F: 6M	Ictal (termination)	8.8 (6.4–11.8)	8.8 (6.4–11.8)	~one week	0	Yes (EEG−21, MEG−275) (4,000 Hz) Connectivity– Source level	fMRI informed MEG effective connectivity (0.5–100 Hz) Beamformer (LCMV), amplitude/ amplitude coupling with canonical	• During the absence seizure, there was a strong coupling between beta and gamma frequencies within the left frontal cortex and between left frontal and right parietal regions. •Strong connectivity between left frontal and right parietal nodes was noted within gamma band.
										frequency bins (1–4, 4–8, 8–12.5, 12.5–30, and 30–59 Hz), multilayer network approach	• Multilayer versatility analysis identified a cluster of network hubs in the left frontal region
**CAE (Resting-state Connectivity)**											
Chavez et al. (125)	CAE	5	NA	Resting state	NA	NA	NA	5	No (MEG−151) (1,250 Hz) Connectivity– sensor level	Graph theory, Linear coherence at sensor level with multi-frequency analysis (<5, 1–15, 15–24, 24–35, >35)	• Compared to a healthy subject, a patient with CAE had richer connectivity and modularity in 5–14Hz
Wu et al. (8)	CAE	13	9F: 4M	Resting state	8 (5.3–11)	NA	13 (0.5–60)	0	No (MEG−275) (6,000 Hz) Connectivity– source level	Beamformer (ASI), Graph theory, Granger causality, correlation analysis at source	• This is the first study to reveal that CAE patients displayed frequency-specific abnormalities in the network pattern during the resting state.
										level with multi-frequency analysis (1–4, 4–8, 8–12,12–30, 30–80, 80–250, 250–500, 500–1,000 Hz)	• Compared to the healthy subject, the network pattern at 1–4 Hz was altered and, at 2 seconds before the first ictal GSWDs, mainly showed a strong connection in the frontal and weakened connection in the anterior-posterior pathway.
**CAE (Difference between interictal and ictal connectivity)**											
Shi et al. (126)	CAE	25	18F: 7M	Interictal and Ictal	7.7 (5–14)	NA	25.52 (1–72)	12	No (MEG−275) (6,000 Hz) Connectivity– source level	Beamformer (ASI), correlation analysis at source level in multifrequency bandwidths (1–4, 4–8, 8–12, 12–30, 30–80, 80–250, 250–500 Hz) (PCC/pC as seed)	• At 4–8, 8–12, magnetic sources of interictal GSWDs mainly located in PCC/pC while in ictal was MFC at 80–120Hz. •During ictal GSWDs, functional connectivity network involving PCC/pC showed strong connections in anterior to posterior pathway at 80–250Hz.
											• During interictal GSWDs, functional connectivity was mostly limited to the posterior cortex region.
Sun et al. (127)^θ^	CAE	22	15F: 7M	Interictal and Ictal	8.5 (5–14)	NA	7.61 (4–13)	7	Yes (EEG−23, MEG−275) (6,000 Hz) Connectivity-Source level	Beamformer (ASI), correlation analysis (1–80 Hz)at source level in two frequency bandwidths (1–30, 30–80 Hz)	• At both frequencies, there was more active source activity location in ictal onset period rather than interictal. •The frontal lobe, temporo-parietal junctions, and parietal lobe became the main active areas of source activity during the ictal period, while precuneus, cuneus, and thalamus were relatively inactive.
**CAE (Treatment response)**											
Tenny et al. (47)^#^	CAE	16	9F: 7M	Pretreatment ictal network	8.8 (6.0–11.8)	8.8 (6.0–11.8)	~1 week	No ASM[Table-fn TN2] f/up at least 2 y	Yes (EEG−21, MEG−275) (4,000 Hz) Connectivity– Source level	fMRI informed MEG effective connectivity (0.1–70 Hz) Beamformer (LCMV), Phase	• Compared to the ETX treatment responder, CAE patients with ETX treatment non-responder had decreased connectivity in the precuneus region with thalamus at the
										slope index in 3 frequency bandwidths (3–4, 13–30, and 30–55 Hz),	delta frequency and increased in the frontal cortex at gamma frequency.
Miao et al. (128)	CAE	25	19F: 6M	Pretreatment ictal network	(4–11)	7.3 (3–10)	NA	No ASM[Table-fn TN2] f/up 36–66 m	No (MEG−275) (300 Hz) Connectivity– source level	Beamformer (ASI) in 2 frequency bandwidth 1–7 Hz and 8–30, Graphic theory—source neural analysis	• Ictal post-DMFC (dorsal medial frontal cortex, including medial primary motor cortex and supplementary sensorimotor area) source at 1–7 Hz or 8–30 Hz were observed in all female patients with LTG non-responder. •The cortico-thalamo-cortical network at 1–7 Hz was changed according to age.
Zhang et al. (129)	CAE	24	19F: 5M	Pretreatment ictal network	10.8 (2–17)	6.29 (4–10)	6.29 (4–10)	No ASM[Table-fn TN2] f/up 12–74 m	No (MEG−275) (6,000 Hz) Connectivity– source level	Beamformer (ASI), Correlation analysis at source level in 6 frequency	• Compared to the ASM (both LTG and VPA) responder, at 8–12and 30–80, the source location of ASM non-responders was mainly in the frontal cortex, mostly the medial frontal cortex.
										bandwidths (1–4, 4–8, 8–12, 12–30, 30–80, 80–250 Hz)	• Nonresponders showed strong positive local frontal connections and deficient anterior and posterior connections at 80–250 Hz.

*y, year; m, month; F, female; M, male; ASM, antiseizure medication; MEG, magnetoencephalography; SAM, synthetic aperture magnetometry; GSWD, generalized sharp wave discharge; sLORETA, standardized low-resolution brain electromagnetic topography; ms, milliseconds; ETX, Ethoxusimide; LCMV, Linear constraint minimum variance; ASI, accumulated source imaging; DICS, Dynamic imaging of coherent sources; LTG, lamotrigine; VPA, valproic acid; PCC, posterior cingulate cortex; pC, precuneus; MFC, medial frontal cortex; CAE, childhood absence epilepsy; NA, no information or not applicable; Y, yes; N, no.*

* *All patients stopped ASM 2 days before MEG.*

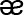
*All patients didn't take any seizure medication at the time of MEG recording and follow up after initiation of ASM.*

** *Same patients were involved in multiple studies.*

θ *Same patients were involved in multiple studies.*

# *Overlapped patients*.

### Source Localization of GSWDs

Multiple studies were published using different source localization techniques to analyze interictal, preictal, and ictal parts of the GSWDs of CAE, as shown in Table 1. Westmijse et al. applied source analysis to ictal GSWDs of human CAE with an average seizure duration of 9s (4–22 s) using non–linear association with the beamformer technique, synthetic aperture magnetometry (SAM). At the onset, sources were localized to cortical brain regions, including left or right frontal, central and parietal, during the spike portion of GSWDs. The sources became generalized during the slow-wave phase (110). A similar finding was reported by Hu et al. using the same technique while analyzing the peak of the spike of GSWD (111). Five out of 13 CAE patients' GSWDs (38.5%) were able to source localized to bilateral frontal regions. The study findings validated the clear cortical sources of activity during the spikes of GSWDs over the bilateral frontal regions and supported the theory of the cortical focus in the generation of generalized epilepsy (111). However, no conclusion could be made regarding deep brain sources (mainly thalamus) due to the limitation of the recording and analysis technique, including the limited high frequency to 70Hz.

Similar to the findings from Rucci et al. (107, 108) and Tenney et al. reported the preictal MEG changes occurred an average of 694 ms before the initial spike of the EEG (112, 114). The same research group (112) aimed to investigate the relative timing of the cortical and thalamus activity in the generation of absence seizures by combining SAM beamformer and standardized low-resolution electromagnetic tomography (sLORETA) to analyze the preictal state, 50 milliseconds (ms) before and after the first ictal spike of ictal GSWDs, in 12 drug-naïve CAE patients. At −50 ms, the seizures were source localized to the frontal cortex, mainly the lateral inferior frontal lobe or thalamus. At the EEG onset (0 ms), focal sources were detected in the frontal cortex with decreased thalamic localization. Following the first ictal spike (50 ms), localization became more widespread. Thus, after the initial frontal and thalamic source, the ictal activity gradually recruited the remaining cortices, i.e., parietal, temporal, and occipital. Later, the same group analyzed the same ictal dataset using time-frequency analysis with different frequency bandwidths (up to 150 Hz) and source localization using sLORETA (113). Tenney et al. were the first to report the network's frequency-dependent nature in CAE (113). The high-frequency oscillations (HFO) 70–150 Hz were localized to the frontal lobe during absence seizures. At lower frequencies, sources were significantly localized to the parietal cortex. Thus, the authors proposed a hypothesis that different oscillations and frequencies favored different types of connections and/or different spatiotemporal levels of information integration. In addition, the finding suggested that the co-occurring frontal and parietal corticothalamic networks interacted to produce a pathological state that contributed to the generation of GSWDs.

The above findings were confirmed by Miao et al. using different beamformer analysis, dynamic magnetic source imaging (dMSI) (115, 116). Miao et al. validated that the source of HFOs (80–500 Hz) in the ictal stage was focal and located in the medial prefrontal cortex (MPFC) compared to the spike portions of the interictal GSWDs, which were widespread (116). In addition, Miao et al. reported that fast ripples (250–500 Hz) were associated with increased seizure frequency (115). Besides, same research group also confirmed the involvement of the default mode network, by reciprocal propagation between medial prefrontal cortex, pre-supplementary motor area, precuneus, and medial occipital cortices, through cortico-cortical pathways *via* medial portion of the brain or cortico-thalamus-cortical pathway *via* thalamus, in the ictal generation and propagation of the seizure activity in CAE (115). Compared to the ictal stage, Xiang et al. studied HFO activity during the interictal stage and compared it with age- and sex-matched healthy controls (117). The authors revealed that patients with CAE had higher odds of interictal HFO activity (either 200–1,000 or 1,000–2,000 Hz) in the parieto-occipito-temporal junction and medial frontal cortices. No significant differences in the deep brain area was reported. Thus, all the above results indicated that CAE had significantly aberrant brain connectivity activity during the interictal as well as the ictal phase, and the above electrophysiological findings could potentially serve as biomarkers for the CAE.

### Connectivity

#### Resting-State

**Figure 1:**
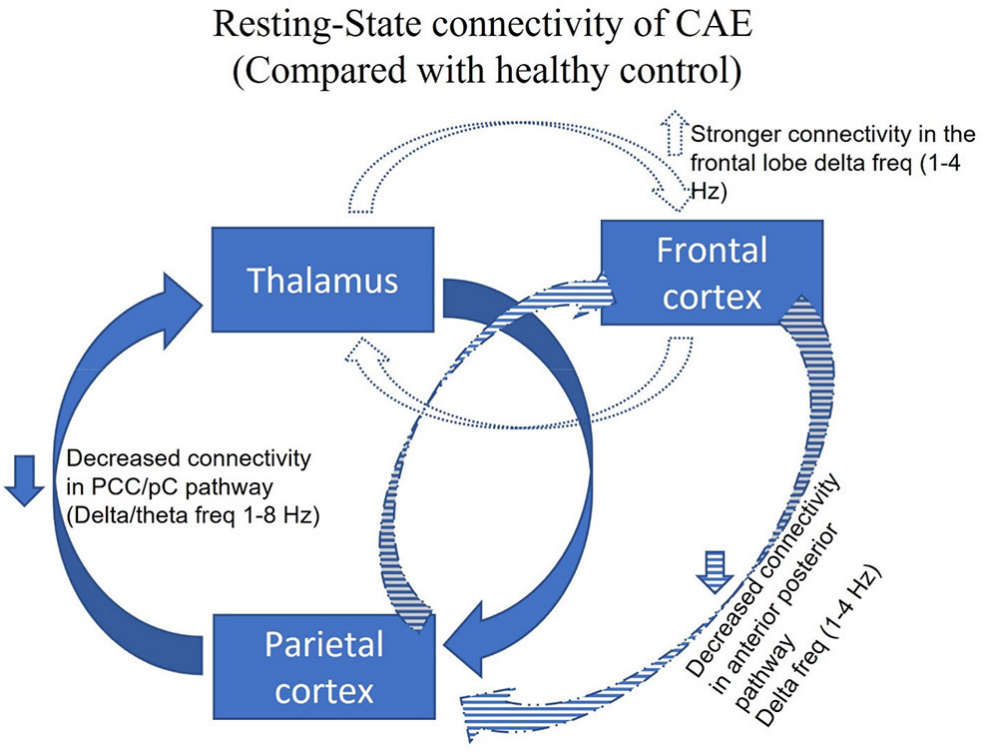
Adapted with permission from Tenney et al. (47). Figure showing the resting-state connectivity in CAE, without 3 Hz GSWDs, compared with the healthy control. The three major brain regions have been identified as responsible for the generalization of the childhood absence seizures (thalamus, frontal cortex, parietal cortex). Given all the data from Wu and colleagues, compared with the healthy control group, strong connectivity in the frontal lobe at 1–4 Hz (blue dotted) and a weakened connectivity in the anterior-posterior pathway was noted within the delta band 1–4 Hz (blue stripped). In the low-frequency band at 1–8 Hz (blue solid), the effective network activity involving the precuneus and posterior cingulate cortex (PCC/pC) was significantly decreased in patients with CAE (8).

Resting-state connectivity of CAE is illustrated in Figure 1. Chavez et al. compared the *modular organization of the brain networks'* resting-state connectivity between CAE patients taking ASMs and normal healthy subjects (125). Increased connectivity with clear modular structures, subsets of units within a network, was noted in the epileptic brain at the extended alpha band (5–14 Hz). Modularity analysis revealed that nodes of epileptic brain networks were abnormally linked to different functional modules in distinct networks compared to the normal healthy subjects. To confirm whether taking ASM might be the contributing factor for altering the resting-state connectivity, Wu et al. studied resting-state connectivity in treatment naïve CAE by constructing effective connectivity (EC) using correlation and Granger causality analysis, and were the first to reveal frequency-specific alteration in EC during the resting state without 3 Hz GSWDs (8). Compared with the healthy control group, strong connectivity in the frontal lobe and weakened connectivity in the anterior-posterior pathway were noted within the delta band (1–4 Hz). In the low-frequency band (1–8 Hz), the effective network activity involving the precuneus and posterior cingulate cortex (PCC/pC) was significantly decreased in patients with CAE (8). The reduced resting functional connectivity in PCC/pC has also been reported in patients with attention deficit disorder and memory impairment (130). Thus, these particular changes may be partially responsible for behavioral and cognitive co-morbidities seen in many patients with CAE (131).

#### Ictal Network Connectivity

Using dynamic imaging of coherent sources (DICS) beamformer, Gupta et al. studied the transitions between interictal, preictal, and ictal periods of absence seizures (within 1s of first ictal GSWD) and confirmed frequency-dependent source localization (119). The consistent appearance of low frequency 2–4 Hz frontal and occipital cortical source was noted before the first generalized spikes, and change in the connectivity networks was noted at the onset of the GSWD, suggesting the increased connectivity from preictal pathologically predisposed network toward the rapidly recruiting synchronous ictal network. Using accumulated source imaging (ASI) beamformer analysis to quantify the network connectivity changes from a preictal to an ictal state, Wu et al. demonstrated that the dynamic changes in neural networks probably resulted from the cortically initialized cortico-thalamic network and analyzed neuromagnetic data as low-frequency (1–80 Hz) and high frequency (80–200 Hz) (120). During the transition period, the predominant neuromagnetic activities were observed at low-frequency (1–80 Hz) dominantly in the frontal and parieto-occipito-temporal cortices, whereas those in the deep brain areas and medial frontal cortex were at a high-frequency band (80–500 Hz) when compared to the interictal period. The EC was mainly over the cortical regions during the interictal period, but when the ictal transition occurred, there was a strong EC between cortex and deep brain areas in both low- and high-frequency ranges. Interestingly, the direction of the EC was predominantly from the cortex to the thalamus in the early ictal period. The same research group conveyed that indeed the rhythmic ictal spiking activity of GSWDs (within 1 s of the ictal spike onset) played a dominant role in the synchronization of the CAE epileptic network at the spike of the GSWDs (at 1–4, 4–8, and 8–12 Hz) which was significantly different from that of the slow-wave of the GSWDs (123). Thus, the dynamically balanced network was distorted primarily by the increased excitatory connections subtending a spike part of the GSWDs. Yet, the connections were mostly excitatory at the high-frequency band (80–250 Hz) regardless of spikes or slow waves. Thus, the authors suggested that abnormal excitatory activity of the entire brain required a local cluster of neurons to initiate the spike discharges, which caused the synchronous hyper-excitability in the epileptic network. Using whole-brain connectivity analysis and linear constraint minimum variance (LCMV) beamformer at the broadband frequency (1–40 Hz), Youssofzadeh et al. tried to reveal the focal components of the absence seizures in effective connectivity (EC) and investigated the network contrast between ictal and preictal period (121). The highly connected brain areas or hubs in the thalami, precuneus and cingulate cortex generally supported a theory of rapidly engaging and bilaterally distributed networks responsible for seizure generation (121).

Not only the ictal transition but also the ictal termination had been studied. Jiang et al. investigated the network changes within the 2 s of ictal termination in drug-naïve CAE using beamformer (ASI) and graph theory connectivity analyses (122). At the low-frequency (1–80 Hz) bands, the activities were predominantly distributed in the frontal and parieto–occipito–temporal junction, whereas sources of HFOs (80–500 Hz) were localized to the medial frontal cortex and deep brain areas (mainly thalamus) during both interictal period and the termination transition. Furthermore, an enhanced positive cortico–thalamic EC was observed around the discharge offset with its direction primarily from various cortical regions to the thalamus (122). Sun et al. re-investigated ictal termination (within the 3 s of transition) of absence seizures and found the transition to be associated with dynamic frequency-dependent changes in the functional connectivity (124). At 1–4, 4–8, and 8–12 Hz, the magnetic source during seizure termination appeared to be consistent over the ictal period and was mainly localized in the frontal cortex and parieto-occipito-temporal junction. At ictal termination, source activity and peak source strength were significantly reduced in the frontal lobe at 12–30 and 30–80 Hz. Thus, the finding from the study, as mentioned above, suggested that the neuromagnetic activity in different frequency bands might play a role in activating or deactivating different cortical networks, such as frontal corticothalamic, parietal corticothalamic, default mode network, etc., and responsible for the pathophysiological mechanism of CAE.

To confirm the hypotheses of whether the interaction of co-occurring networks at distinct frequencies interact through cross-frequency coupling mechanism effects, Tenney et al. complemented neuromagnetic signal analysis, beamformer (LCMV), and cross-frequency canonical analyses with fMRI to increase the spatial resolution and analyze cross-frequency coupling (CFC) (48). The fMRI informed MEG effective connectivity (EC) (spatial map of the ictal network was defined using the fMRI and used as *a prior* for MEG connectivity) study showed beta/gamma CFC and within frequency coupling in frontoparietal and frontofrontal regions during the CAE seizures. Strong coupling between beta and gamma frequencies within the left frontal cortex, and between left frontal and right parietal regions were observed. There was also strong connectivity between left frontal and right parietal nodes within the gamma bands. Multilayer versatility analysis showed that a cluster of network hubs in the frontal regions and thus frontal cortical regions were critical for absence seizure generation (48). Thus, all the findings from the ictal connectivity studies consistently show different cross-frequency coupling or distinct frequency-dependent activation and deactivation of cortical network initiation followed by abrupt synchronization between cortical and subcortical structures in the generation, propagation, and the termination of the CAE seizure, which further supports the *cortical focus theory*.

#### Difference Between Ictal and Interictal Connectivity

**Figure 2:**
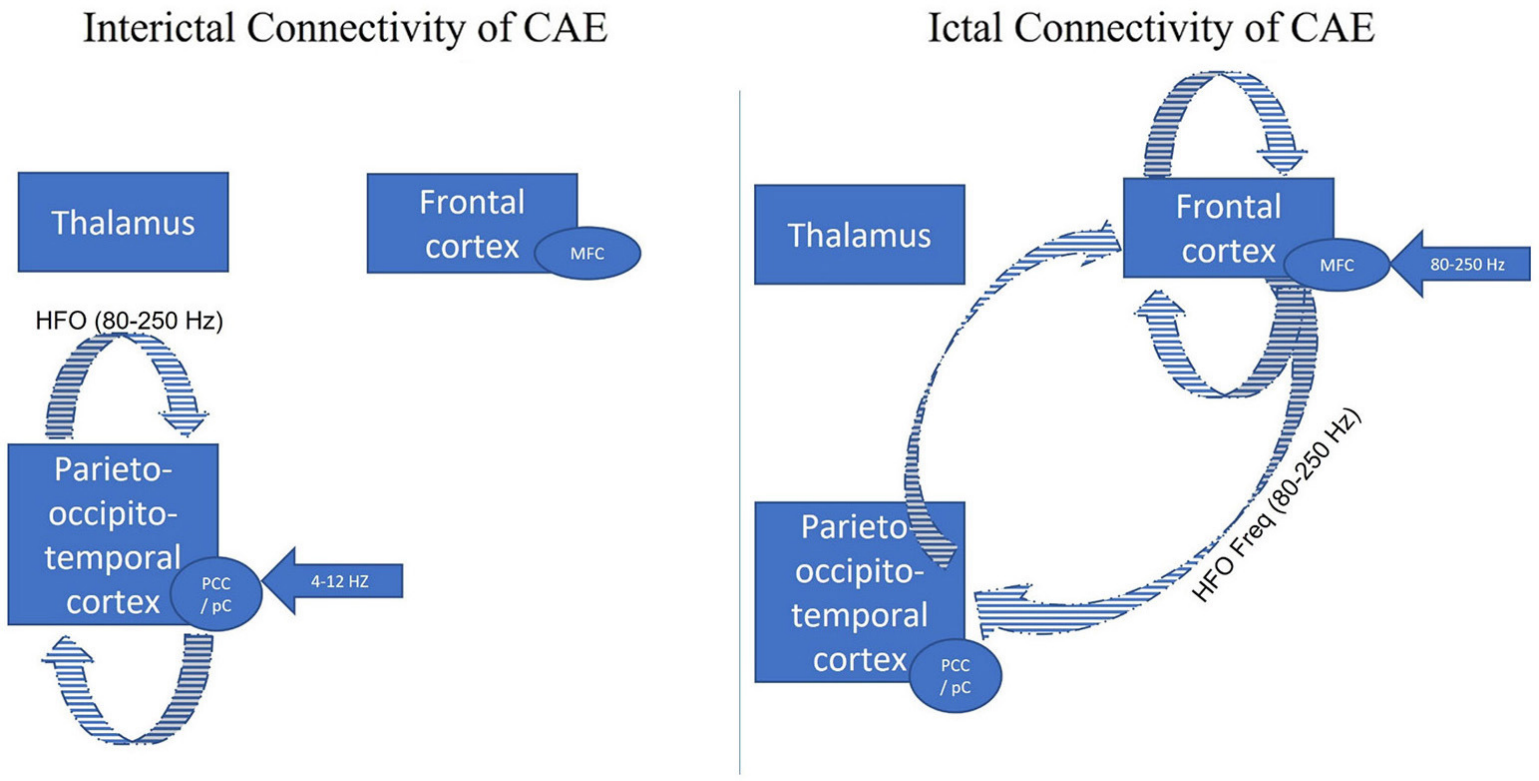
Adapted with permission from Tenney et al. (47). Figure comparing the interictal (GSWDs <4 s) and ictal connectivity (GSWDs > 10 s) in CAE. The three major brain regions have been identified as responsible for the generalization of the childhood absence seizures (thalamus, frontal cortex, posterior cortex). Given all the data from Shi et al. (126) the low frequency (4–8 Hz and 8–12 Hz) magnetic sources were mainly localized within the posterior cingulate cortex and precuneus (PCC/pC) during the interictal state (blue arrow). The high frequency (80–250 Hz) magnetic components of the ictal GSWDs were mainly localized in the medial frontal cortex (blue arrow). In terms of connectivity (using posterior cingulate and precuneus (PCC/pC) as the seed), there were strong connections in the anterior-posterior pathway, mainly the frontal cortex during the ictal GSWDs (blue stripped). In contrast, the connections were mostly limited to the posterior cortex region at 80–250 Hz during interictal GSWDs (blue stripped). Thus, there were significant disparities in the source localization between ictal and interictal GSWDs.

Ictal and interictal GSWD connectivity were studied using ASI beamformer and correlation analyses to investigate the clinical ictal symptoms related to the ictal CAE epileptic network and illustrated in Figure 2. Shi et al. investigated the differences between the interictal GSWDs (<4 s) and ictal GSWDs (>10 s) in CAE (126). The low frequency (4–8 Hz and 8–12 Hz) magnetic sources were mainly localized within the posterior cingulate cortex and precuneus (PCC/pC) during the interictal state. The high frequency (80–250 Hz) magnetic components of the ictal GSWDs were mainly localized in the medial frontal cortex. In terms of connectivity (using posterior cingulate and precuneus (PCC/pC) as the seed), there were strong connections in the anterior-posterior pathway, mainly the frontal cortex during the ictal GSWDs. In contrast, the connections were mostly limited to the posterior cortex region at 80–250 Hz during interictal GSWDs. Thus, there were significant disparities in the source localization between ictal and interictal GSWDs. The low-frequency activation in the PCC/pC during the interictal period might be related to maintaining consciousness during the interictal GSWDs. Shi et al. concluded that weakened network connections during the interictal GSWD might be in favor of preventing overexcitability and relates to the termination of GSWDs (126). Thus, the finding concurs with the conclusion made by Wu et al. (8). There is reduced resting functional connectivity in PCC/pC patients with CAE in not only interictal but also resting state.

To confirm the findings, the same research group (127) studied the functional connectivity reorganization of the brain regions in both interictal without GSWD (30 s of the interictal period without GSWDs at least 60 s from the ictal period) and ictal GSWD network using two frequency band activities (1–30 Hz and 30–80 Hz). Compared to the interictal period, frontal, temporoparietal, and parietal regions were more active during seizures. On the contrary, the precuneus, the cuneus, and the thalamus were relatively silent during the ictal period compared with the interictal period. The differences in source localization between ictal and interictal states were reported, regardless of seizure duration, seizure frequency, or the age of epilepsy onset. Thus, the available data suggests the role of different frequency-dependent initial cortical involvement, most importantly in the frontal region, with predisposing hyper-excitable corticothalamic synchronization in the generation of the CAE.

#### Treatment and Prognostic Biomarker

Miao et al. reported that the presence of fast ripples (250–500 Hz) HFO in absence seizures was associated with increased seizure frequency (116). Similarly, Tang et al. studied whether the HFO in drug-naïve CAE was related to seizure severity and reported that the strength of ictal HFO (200–1,000 Hz) was significantly correlated with the severity of the absence seizures measured by the number of daily seizures, therefore a potential prognostic biomarker (118). Sun et al. later reported that the ictal peak source strength in the 1–4 Hz range was negatively correlated with the ictal duration of the seizure, whereas at 30–80 Hz, there was a positive correlation with the course of epilepsy (124). Yet, both studies were not able to draw major conclusions due to the limitation of a cross-sectional study (116, 118).

**Figure 3:**
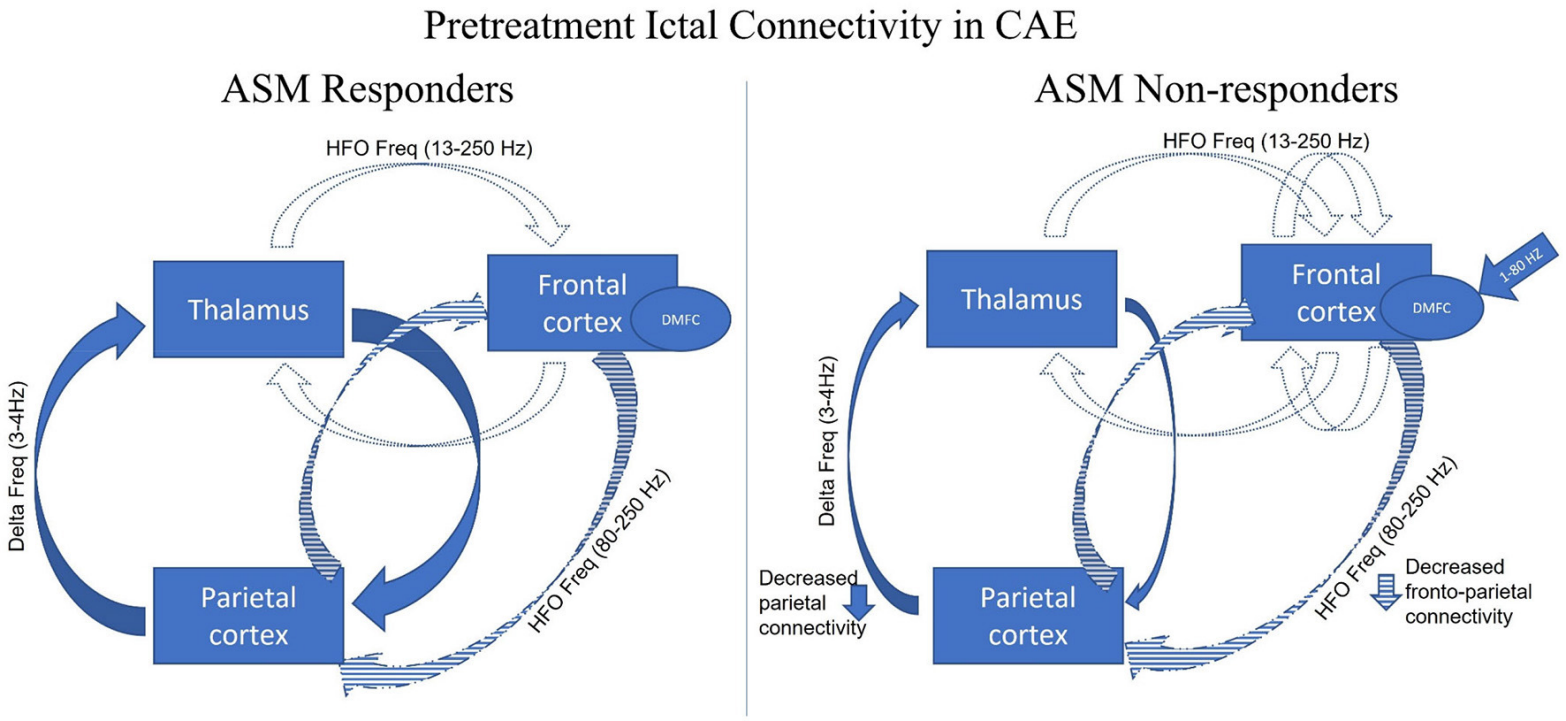
Adapted with permission from Tenney et al. (47). Figure showing pretreatment ictal connectivity analysis of the antiepileptic (ASM) (ethosuximide, lamotrigine, and valproic acid) responder and non-responder in CAE. The three major brain regions have been identified as responsible for the generalization of the childhood absence seizures (thalamus, frontal cortex, parietal cortex). Given all the findings collected from Tenny et al. (47), Miao et al. (128), and Zhang et al. (129), parietal cortico-thalamic network at the low frequencies (blue solid) and a co-occurring frontal corticothalamic network at higher frequency (blue dotted) and anterior-posterior/frontoparietal network at higher frequency (blue stripped). Compared to the responder, ASM non–responders have ictal neuromagnetic sources at 1–80 Hz localized to the dorsomedial frontal cortex (DMFC) (blue arrow). In addition, the ASM non–responders have decreased parietal cortico-thalamic at 3–4 Hz and frontoparietal network connectivity at 80–250 Hz with increased frontal local connectivity at 13–250 Hz.

Thus, a couple of studies were conducted in which patients with drug-naive CAE underwent a MEG recording at the time of diagnosis (or within 1 week of diagnosis) and followed up for at least 1 year. The difference in the pretreatment ictal connectivity in patients with CAE was studied in response to ASMs treatment (responder vs. non-responder) and illustrated in Figure 3. Tenney et al. used fMRI-informed MEG effective connectivity to study prognostic biomarkers prospectively in drug-naive CAE patients with a follow-up for at least 2 years after starting the ethosuximide (ETX) (47). Pretreatment connectivity demonstrated the strongest connections in the thalamus and posterior head regions (parietal, posterior cingulate, angular gyrus, precuneus, and occipital) at low frequency (delta 3–4 Hz) and the co-occurring frontal cortical thalamic network at the high frequencies (beta/gamma 13–55 Hz). ETX non-responders' pretreatment connectivity decreased in the precuneus region and increased in the frontal cortex compared to ETX responders. This increased frontal cortical connectivity may be a potential prognostic biomarker of drug-resistance. Miao et al. also studied the responders and non–responders to the two established ASMs, lamotrigine (LTG) and valproic acid (VPA), using a beamformer (ASI) (128). In six LTG-non-responders CAE patients, ictal source locations were noted in the posterior-dorsal medial frontal cortex (post-DMFC including medial primary motor cortex and supplementary sensorimotor area) at 1–7 Hz or 8–30 Hz but not in 9 LTG responders, regardless of the age of onset and the seizure frequency. In addition, the authors suggested that ictal post-DMFC source localization could be suggestive of a biomarker for predicting LTG non–responsiveness. Zhang et al. replicated the same findings in CAE patients using the same beamformer technique (ASI) (129). The source localization of the ASMs non-responders was mainly in the frontal cortex at 8–12 and 30–80 Hz, especially the medial frontal cortex at alpha frequency. The non-responders showed strong positive local frontal connections and deficient anterior and posterior connections at 80–250 Hz. Thus, while it is likely that no one single mechanism can explain the pharmacologic responsiveness, ASM non-responders had more source localized within the dorsomedial frontal regions with decreased anterior-posterior network connectivity. At this time, the available preliminary data shows promising results in prognosticating response to ASM, but further studies with a larger sample size as well as comparing types of ASM non-responders are warranted to study the causality association.

Thus far, neuromagnetic source localization identifies three major brain areas which are thought to be responsible for the generation, propagation, and termination of CAE GSWDs: frontal cortex, parietal cortex, and thalamus with earlier cortical sources (more than 500 ms), supporting the concept of *cortical focus theory*. In addition, the current MEG literature suggests that the pathophysiology of GSWD in the absence seizure is a reflection of the co-occurring (excitation or inhibition) network(s) pathology rather than dysfunction in one particular brain area. For instance, frontal hyperexcitability and parietal deactivation involving intact but altered EC networks, such as corticothalamic, corticocortical and default mode networks, triggering the rapidly generalized epileptic discharges.

## Juvenile Absence Epilepsy (JAE)

Although JAE and CAE share many similar clinical characteristics, CAE absence seizure has more pronounced impairment of consciousness, and tonic-clonic seizures are less common than JAE. In terms of EEG, GSWDs of JAE are usually a higher frequency at 4–5 Hz. In terms of prognosis, JAE has a slightly worse prognosis when compared to CAE (132). Studies published on JAE are illustrated in Table 2.

**Table 2:** Showing all the published study characteristics and main outcomes on juvenile absence epilepsy and combined absence epilepsy.

**Article name**	**Type of genetic epilepsy**	**No. of patients included in study**	**No. of female (F): No. of male (M)**	**Study state of genetic epilepsy**	**Mean age at the time of MEG recording (range) (y)**	**Mean age of epilepsy onset (range) (y)**	**Duration of epilepsy (range) (m)**	**No. of pt. on ASM**	**Yes and No for the Simultaneous MEG/EEG recording** **No. of EEG, MEG sensor with sampling rate of the MEG recording** **Source or sensory level (for the connectivity study only)**	**Type of analysis with Analyzed MEG Frequency bandwith**	**Main result**
**JAE**											
Amor et al. (133)	JAE	5	4F: 1M	Ictal	23.4 (18–31)	NA	NA	2	Yes (EEG−64, MEG−151) (1,250 Hz) Connectivity– source level	Analytical wavelets transform (0.2–25 Hz), phase-locking	• At the ictal onset, there was reproducible sequence of changes in the cortical network (i) long-range desynchronization, (ii) increased local synchronization, and then followed by (iii) increased long-range synchronization.
Sakurai et al. (134)	JAE	5	2F: 3M	Ictal/GSWDs	27.2 (21.38)	NA	16 (12–26)	4	Yes (EEG−21, MEG−204) (600 Hz)	Dynamic statistical parameter mapping (dSPM) (0.5–400 Hz)	• Initial activation of the spike of GSWDs was noted over focal cortical regions, the medial prefrontal activation followed by activation of posterior cingulate and precuneus, resulting in the involvement of disruption of default mode network.
**Combined CAE and JAE**											
Rozendaal et al. (135)	1JAE,6CAE	7	4F:3M	Interictal/ GSWDs	9.4 (7–14)	6.4 (5–12)	NA	7	No (MEG– either151 or 275) (1,200 Hz)	Equivalent dipole model (ECDs)(3–70 Hz)	• ECDs were localized most often on frontal, central, or parietal origin in either right or left hemisphere (with stable locations on averages of these discharges)
Gadad et al. (136)	8JAE, 12CAE	20	10F:10M	Ictal/ GSWDs	11.15 (7–30)	8.1 (2-19).	32.4 (18–72)	5	Yes (EEG −23, MEG−306) (2,000 Hz)	LORETA (1–70 Hz)	• The most common involved regions were caudate, cingulate, lentiform nucleus, and thalamus at the onset of all groups of GSWDs (1 s, >1s but <9 s or >9 s). •During the propagation, most frequent locations of sources were at limbic and frontal lobes with either lateralized and localized, and then source propagated to front-limbic structures at the offset, irrespective of the duration of GSWD and subtype of absence epilepsy.

*y, year; m, month; F, female; M, male; ASM, antiseizure medication; MEG, magnetoencephalography; SAM, synthetic aperture magnetometry; GSWD, generalized sharp wave discharge; LORETA, low-resolution brain electromagnetic topography; ms, milliseconds; ETX, Ethoxusimide; LCMV, Linear constraint minimum variance; ASI, accumulated source imaging; DICS, Dynamic imaging of coherent sources; LTG, lamotrigine; VPA, valproic acid; PCC, posterior cingulate cortex; pC, precuneus; MFC, medial frontal cortex. CAE, childhood absence epilepsy; JAE, juvenile absence epilepsy; JME, juvenile myoclonus epilepsy; TCS, tonic-clonic seizure; IGE, idiopathic generalized epilepsy; GGE, genetic confirmed generalized epilepsy; NA, no information or not applicable; Y, yes; N, no*.

Amor et al. explored the spatio-temporal dynamics of interactions within and between widely distributed cortical sites using MEG in patients with JAE (133). At the ictal onset, localized phase synchronization in multiple frontal and precentral areas was recorded, and the activity preceded the first ictal EEG GSWDs by 1.5 s. The analyses revealed a reproducible sequence of changes in the cortical network: (1) long-range desynchronization, (2) increased local synchronization, and (3) increased long-range synchronization. However, both local and long-range synchronization displayed different spatio-temporal profiles, but the cortical projection within the initiation time window (500 ms before the first ictal GSWDs) overlapped multifocal fronto-central regions, such as left frontomedial, frontopolar, right orbitofrontal, and right latero-central regions. Sakurai et al. studied the source analysis of the GSWDs in 5 patients with JAE using a dynamic statistic parametric mapping (dSPM) approach (134). The researchers reported that the initial activation of the GSWDs appeared in the focal cortical region with strong activation over the medial prefrontal activation followed by posterior cingulate and precuneus in 3 out of 5 patients simultaneously right after the medial prefrontal activation (134). The area mentioned above involved the default mode network at the onset of the GSWD, and thus it wasn't random diffuse cortical involvement but rather a selective cortical network, particularly the default mode network.

## Combined Absence Epilepsy (Childhood and Juvenile Absence Epilepsy)

Studies published on combined absence epilepsy (CAE and JAE) are illustrated in Table 2. Rozendaal et al. attempted to compare the interictal and ictal periods in absence epilepsy (6CAE and 1JAE) using the SECD model, and source localizations were most often frontal, central, and parietal regions in either left or right hemisphere (135). The spatiotemporal assessment of the interictal epileptiform discharges (IEDs) indicated a stable localization of the averaged discharges, indicating a single underlying cortical source. Using LORETA, Gadad et al. studied the source analysis of the GSWDs at the onset, during, and offset of the GSWDs based on the duration of GSWDs and divided into three groups: GSWDs lasting 1 s, more than 1 s but less than 9.9 s, and equal to more than 10 s (136). The authors reported that the most common involved regions were caudate, cingulate, lentiform nucleus, and thalamus at the onset of all average discharges. Thus this observation substantiated the previously documented thalamo-cortico-stratum involvement in the absence of epilepsy (22, 23). During the propagation, the most frequent localization of sources were at limbic and frontal lobes, and these sources propagated to fronto-limbic structures at the ictal offset, irrespective of the duration of GSWD and subtype of absence epilepsy. The finding indicated the restricted/sustained network circuitry in fronto-limbic network involvement in origination and propagation of GSWDs until the disruption and inhibition. No significant difference in the source localization or network involvement was noted between CAE and JAE.

## Juvenile Myoclonus Epilepsy (JME)

### Source Localization of GSWDs

Studies published on JME are illustrated in Table 3. Kotini et al. reported 2 adults with JME using the multiple signal characterization (MUSIC) algorithm and showed that the dipolar sources of the peak of GSWDs were localized at the cerebellar vermis with an extension up to the occipital region (137). Instead of analyzing at peak of the spike, Gadad et al. studied the source analysis of average GSWDs in three different spike phases: onset (upward phase of the spike from the baseline), peak, and offset (trailing edge of the spike) using LORETA in 20 patients with JME. At the onset of the GSWDs, the majority of the neuromagnetic sources were localized to sublobar regions (31% of localized discharges) defined as insula, caudate, claustrum, lentiform nucleus, and thalamus, followed by limbic region (22%), frontal (22%) and temporal lobe (11%). At the peak of the discharges, the sources were localized to the frontal lobe (45%), followed by sublobar regions (23%) (mainly lentiform nucleus). At the offset of the discharges, the sources were localized to the sublobar region (28%) (mainly caudate), followed by limbic (24%) and frontal regions(18%) (138). Therefore, the available evidence suggests an overall synchronous on and off interaction of cortico-subcortical structures in generating and propagating the epileptiform discharges in JME.

**Table 3:** Showing all the published study characteristics and main outcomes on juvenile myoclonic epilepsy and combined genetic epilepsy.

**Article name**	**Type of genetic epilepsy**	**No. of patients included in study**	**No. of female (F): No. of male (M)**	**Study State of genetic epilepsy**	**Mean age at the time of MEG recording (range) (y)**	**Mean age of epilepsy onset (range) (y)**	**Duration of epilepsy (range) (m)**	**No. of pt. on ASM**	**Yes and No for the Simultaneous MEG/EEG recording** **No. of EEG, MEG sensor with sampling rate of the MEG recording** **Source or sensory level (for the connectivity study only)**	**Type of analysis with analyzed MEG Frequency bandwith**	**Main result**
**JME (Interictal/Ictal GSWDs)**											
Kotini et al. (137)	JME	2	1F: 1M	GSWDs	25.5 (22/29)	17.5 (17/18)	96 (60/132)	2	No (EEG−18, MEG−122) (256 Hz)	Multiple signal characterization (MUSIC) algorithms (0.3–40 Hz)	• Dipolar sources of GSWDs were localized at the cerebellar vermis with extension upto the occipital region
Gadad et al. (138)	JME	20	10F: 10M	GSWDs	23.5 (NA)	16 (NA)	91.2	7	Yes (EEG−23, MEG −306) (2,000 Hz)	LORETA (1–70 Hz)	• At the onset of the GSWDs discharges, the sources were localized to sublobar regions, defined as insula, caudate, claustrum, lentiform nucleus, and thalamus, followed by limbic region, frontal and temporal lobe. •At the peak of the discharges, the sources were localized to the frontal lobe, followed by the sublobar regions (mainly lentiform nucleus).
											• At the offset of the discharges, the sources were localized to the sublobar region(mainly caudate), followed by limbic and frontal regions.
**JME (Task-Specific)**											
Hamand et al. (139)	JME	12	9F:3M	Task-Specific Resting-state	24.1 (18–37)	13.8 (8–17)	NA	12	No (MEG−275) (1,200 Hz)	Beamformer (SAM) (15–30hz, 40–60Hz, 60–90 Hz)	• Compared to healthy control, patients with JME had significantly reduced pre-movement beta event-related desynchronization in the motor task.
De León et al. (140)	JME	1	1M	Task Specific Reflex Seizure	29	8	252	1	Yes (EEG−64, MEG−305) (1,000 Hz)	Forward and inverse modeling, weighted minimum-norm estimation (wMNE) (0.1–330 Hz)	• Source localization of ictal GSWDs was localized to the premotor frontal cortex.
**GGE (Interictal GSWDs)**											
Stefan et al. (141)	IGE (2 JME, 4 AE, and 6 AE-TCS)	7	4F,3M	GSWDs (Spike)	27.86 (17–42)	NA	NA	6	Combined (5 patients has simultaneous MEG-EEG recording, 2 patients has only MEG recording) (EEG−32, MEG-two sensor system with 37 first order gradiometers) (N/A on sampling rate)	Equivalent dipole model (Single dipole analysis/Single moving dipole), Beamformer (normalized scanning analysis) (N/A on frequency band-width)	• In all patients, source analysis showed most often involvement of frontal, peri-insular, and subcortical/thalamic areas in addition to the unilateral frontal accentuation. •In JME and Myoclonic absence epilepsy, source analysis showed central and premotor regions whereas prefrontal accentuation in absence epilepsy.
**JME (Resting-state connectivity)**											
Routley et al. (142)*	JME	26	19F: 7M	Resting-state	28.5 (18–48)	14 (17–24)	181 (33-488)	26	No (MEG−275) (600 Hz) Connectivity—source level	Beamformer (LCMV)(1–150 Hz), Graphic theory, source-level analysis with correlation analysis with different frequency	• Compared to healthy control, patients with JME had increased connectivity in the theta band in the posterior head region and decreased connectivity in the beta band in the sensorimotor cortex
										bandwidth (1–4, 4–8, 8–13, 13–30, 40–60 Hz)	
Krzemiński et al. (143)*	JME	26	19F: 7M	Resting-state	28.5 (18–48)	14 (7–24)	181 (33-488)	26	No (MEG−275) (600 Hz) Connectivity—source level	Graphic theory, source-level analysis with pairwise maximum entropy model (pMEM) with different frequency bandwidth (4–8, 8–12, 13–30, 350–60 Hz)	• Compared to healthy control, JME patients showed fewer local energy minima and elevated energy values for frontoparietal networks within theta, beta, and gamma bands.
Lopes et al. (144)*	JME	26	19F: 7M	Resting-state	28.5 (18–48)	14 (7–24)	181(33-488)	26	No (MEG−275) (600 Hz)	Beamformer (LCMV), Canonical mathematical model of ictogenicity at alpha band	• Compared to healthy control, patients with JME had a higher propensity to generate seizures. The BNI classification accuracy was 73%
										8–13 Hz (Brain network ictogenicity BNI)	
**GGE (Resting State Connectivity)**											
Elshahabi et al. (145)	IGE (5IGE-TCS, 4CAE,2JAE, 1 JME, 1 UN)	13	9F:4M	Resting-state	38.6 ± 15.8	15.5 (4–48)	NA	12	No (MEG−275) (3,906.2 Hz) Connectivity –source level	Beamformer (DICS), Graphic theory, source analysis at different frequency bandwidths (0–4, 4–8, 8–12, 12–20, 21–29, 35–45 Hz)	• Compared to the healthy control, patients with IGE had a widespread increase in connectivity, mainly in the motor network, mesio-frontal and temporal cortex.
Stier et al. (146)	GGE (5CAE,6JAE, 5JME, 4 TCS and 5GGE)	25	16F: 9M	Resting state	25 (22–37)	15 (10–17)	204 (96-288)	NA	No (MEG−275) (585.9 Hz) Connectivity—sensor level	Beamformer (DICS), Graphic theory, the imaginary part of coherency, source analysis at different	• Compared to the healthy control, patients with generalized epilepsy showed widespread increased functional connection at the theta and gamma frequency band and power in the delta and gamma frequency band.
										frequency bandwidths (0–4, 4–8, 8–12, 12–20, 21–29, 32–48 Hz)	• Compared to normal control, siblings without epilepsy also had significantly increased network connectivity, predominantly in beta frequencies, representing an endophenotype of GGE
**Difference between healthy control, generalized epilepsy, and focal frontal epilepsy**											
Niso et al. (147)	JME	15	9F: 6M	Resting state	27 (20–46)	NA	NA	15	No (MEG−306) (1,000 Hz) Connectivity—sensor level	Graphic theory, phase lag value at sensor level analysis (0.5–40 Hz) with multi-frequency bandwidth (0.1–4, 4–8, 8–12,12–20, 20–28, 28–40)	• Generalized epilepsy showed higher spectral power for all the frequencies over the widespread sensors except the alpha band, whereas frontal lobe epilepsy showed higher relative power in the beta band bilaterally over the frontocentral sensors. •In generalized epilepsy, network connectivity showed greater efficiency and lower eccentricity than the control subjects at high-frequency bands.
											• Frontal focal epilepsy patients showed reduced eccentricity for theta band over the frontotemporal and central sensors.
Li Hegner et al. (148)	IGE (8 IGE-TCS, 2 CAE, 3 JME, 3 AE-TCS)	17	12F, 5M	Resting state	33.2 (18–63)	15.3 (6–47)	NA	15	No (MEG−275) (586 Hz) Connectivity—source level	Beamformer (DICS), Graphic theory, the imaginary part of coherency, source analysis at different frequency bandwidths (0–4, 4–8, 8–12, 12–20, 21–29, 30–46 Hz)	• Compared to healthy control, both focal frontal and generalized epilepsy patients showed widespread increased functional connectivity. •Compared to focal epilepsy, generalized epilepsy patients had increased network connectivity in bilateral mesio-frontal and motor regions.

*y, year; m, month; F, female; M, male; ASM, antiseizure medication; MEG, magnetoencephalography; SAM, synthetic aperture magnetometry; GSWD, generalized sharp wave discharge; LORETA, standardized low-resolution brain electromagnetic topography; ms, milliseconds; LCMV, Linear constraint minimum variance; ASI, accumulated source imaging; DICS, Dynamic imaging of coherent sources; CAE, childhood absence epilepsy; JAE, juvenile absence epilepsy; JME, juvenile myoclonus epilepsy; TCS, tonic-clonic seizure; IGE, idiopathic generalized epilepsy; GGE, genetic confirmed generalized epilepsy; NA, no information or not applicable; Y, yes; N, no.*

* *Same patients*.

### Network Connectivity

#### Resting-State

Three publications from the same research group reported and studied the 26 JME patients taking ASM using three different neuromagnetic source localization and connectivity techniques (142–144). Routley et al. studied resting-state functional connectivity in 26 patients with JME and reported that the altered resting-state connectivity could be a neuropathophysiological hallmark or potential diagnostic biomarker for JME. Compared to the healthy control group, there was overall increased connectivity in the posterior head regions in theta and alpha bands, and decreased connectivity in the pre and post-central brain region in beta bands. The reported increased connectivity in the posterior theta-frequency band might be associated with long-range connections affecting attention and arousal. The decreased beta band sensorimotor connectivity might be related to the resting state sensorimotor network and seizure-prone states in JME (142). Using a pairwise maximum entropy model, Krzeminski et al. studied the divergent oscillatory power in different networks: frontoparietal network (FPN) (ROIs: middle frontal gyrus, pars triangularis, inferior parietal gyrus, superior parietal gyrus, and angular gyrus), default mode network (ROIs: orbitofrontal cortex, precuneus, posterior cingulate, anterior cingulate and angular gyrus), and sensorimotor network (ROIs: supplementary motor area, precentral gyrus, and postcentral gyrus). Compared with the healthy control group, JME patients had fewer local energy minima and had elevated energy values for the FPN within theta, beta, and gamma bands during the resting state. No significant changes were noted between the default mode and sensorimotor networks using this method (143). Similar to the findings seen in CAE, these results highlighted the involvement of FPN in the pathophysiology of the JME.

Lopes et al. studied the same cohort of JME patients to investigate computational biomarkers using brain network ictogenicity (BNI), a computational modeling method, to generate the synthetic activity fluctuating between resting and seizure states (144). The higher values of the BNI represent a higher inherent propensity of the brain to generate seizure activity. Lopes et al. reported that patients with the JME had higher BNI values than healthy controls, and sensitivity was reported to be 0.77, and specificity was 0.58, with an area under the curve was 0.72 (144). But the model couldn't be generalized beyond JME as there was no study comparing other types of epilepsy.

#### Task-Specific Cortical Modulation

Hamandi et al. studied the resting state response in task-specific cortical modulation in occipital and sensorimotor cortices in JME compared to healthy control individuals (139). The authors reported that patients with JME had significantly reduced pre-movement beta event-related desynchronization in ipsi- and contralateral sensorimotor areas compared to controls, before and during the transient movement of motor tasks. There was no difference between epileptic and health patients in movement-related gamma synchronization and post-movement beta rebound. In addition to the physical motor task, De León et al. reported a case of mental calculation induced seizure in a patient with JME where the source was localized to the right premotor frontal cortex using the weighted minimum norm estimates (140). Similar to the result presented by Routley et al. and Krzeminski et al. with decreased sensorimotor connectivity, the current two task-specific JME patients suggested an abnormality in motor planning in JME likely related to the altered resting-state sensorimotor network and seizure-prone states in the JME (142, 143).

## Combined Genetic/Idiopathic Generalized Epilepsy

Stefan et al. studied a total of 7 patients with various idiopathic generalized or genetic confirmed generalized epilepsy (IGE/GGE) using beamformer. After analyzing spike-wave bursts in all patients and single spikes in 6 patients, source analysis showed most frequently involved regions were the left or right frontal (mainly mesial and bilateral frontal areas), peri-insular, and subcortical/thalamic areas. In addition, all patients had unilateral frontal accentuation of the activity. In three patients, two with JME and one with myoclonic absence epilepsy, sources were mainly present in the central and premotor regions (141). Thus, the authors concluded that in contrast to pure focal epilepsy, the distribution of the GSWD is not restricted to one hemisphere but a predominant region with additional oscillating connectivity within the thalamocortical network system.

Elshahabi et al. studied the resting-state connectivity of 13 patients with various types of IGE/GGE using beamformer and graph theoretical network analysis. Compared to normal controls, the patients with IGE/GGE had more pronounced motor network connectivity, mainly superior frontal gyrus, precentral, postcentral gyri, temporal cortex, and cerebellum. The authors also found significantly increased regional connectivity in the temporal lobe (superior and inferior temporal gyri) and insula (145). However, no conclusion could be made given that the study was performed on various IGE/GGE types and the limitation of the sub-cortical localization using a particular technique.

Stier et al. studied a total of 25 patients with GGE. Compared to normal healthy individuals, there was an increased functional connectivity at the multi-frequencies level in patients with GGE. Compared to normal controls, siblings without epilepsy also had significantly increased network connectivity, predominantly in beta frequencies. Compared to the healthy siblings of GGE, the increased beta connectivity patterns in GGE patients were less concordant, followed by functional connectivity in theta and delta frequency bands. Thus, the authors proposed that increased interictal MEG power and connectivity in frontocentral and temporo-parietal cortical regions were potential hallmarks of GGE (146). In addition, changes in these network characteristics were likely driven by the genetic factor and not by the disease process or medication effect (146).

## Difference in Resting-State Functional Connnectivity Between Focal (Frontal) and Generalized Epilepsy

Using the fMRI connectivity analysis, it has been reported that patient with frontal lobe epilepsy has variable connectivity, either reduced or increased, various resting-state networks when compared to healthy pediatric and adult population (149–152). Still, there is limited literature investigating the resting-state fMRI functional connectivity comparing frontal lobe epilepsy with generalized epilepsy. A few publications on MEG resting-state functional connectivity in temporal lobe epilepsy are available, but data on frontal lobe epilepsy remains scarce. Herein, we would like to describe available neuromagnetic data in comparing the resting state connectivity between focal and generalized epilepsies.

### Difference Between JME and Frontal Lobe Epilepsy

Niso et al. studied the resting-state functional connectivity of patients with frontal lobe epilepsy (FLE), generalized epilepsy (JME), and healthy individuals. Using power spectral analysis and graph theory assessed by phase synchronization measured with functional connectivity, the distribution of power and topographic changes (activation or deactivations) differed among all three groups. An increased total power indicated local synchronization. Those with JME had a higher total power for all frequencies except alpha band over a widespread set of sensors, whereas the FLE group showed higher relative power in the beta band bilaterally in the frontocentral sensors; i.e., regional specific around the epileptic focus. The authors found that functional networks from generalized epilepsy had greater efficiency and lower eccentricity than control subjects for higher frequency bands without a clear topography. Functional networks in FLE exhibited only reduced eccentricity over the frontotemporal and central sensors relative to the networks from controls (147). Thus, JME and FE groups represent a characteristic pattern of changes as compared to control.

### Difference Between IGE/GGE and Frontal Lobe Epilepsy

Li Hegner et al. studied functional MEG connectivity using graph theory and coherency between focal and generalized epilepsy during resting state (with the absence of spikes or GSWDs) and found significant differences in network connectivity. Increased network connectivity was noted in bilateral mesio-frontal and motor regions in patients with IGE/GGE (148). Thus, the difference in the topography of resting-state functional connectivity in the mesio-frontal region in IGE/GGE may be a specific diagnostic biomarker.

## Conclusion and Future Perspectives

In summary, with the advanced signal processing techniques combined with excellent temporal resolution properties of MEG, the cerebral neuromagnetic sources of GSWDs can be recorded and analyzed with millisecond resolution (153). The recording and post-processing associated with earlier MEG recording on GSWDs, especially using the SECD model, has several limitations, including deep brain structures, signal analysis of high-frequency oscillation, frequency-dependent network changes, etc. Later recordings using various advanced methodologies (various types of the beamformer, LORETA, pMEM, mathematical brain modeling, frequency coupling, etc.) advance our understanding not only of the potential pathophysiology of generalized epilepsy but also shed light on potential diagnostic, therapeutic and prognostic biomarkers of generalized epilepsy.

This review clearly illustrates the transition from focal neuromagnetic source analysis to network-based analysis using different frequency bandwidths involved in the generation, propagation and termination of the generalized spikes in various types of GGE. Earlier neuromagnetic analysis data focused on one particular brain structure, but recent literature points out that both cortical and subcortical structures are equally important in addition to the intact connectivity between various corticocortical and cortico-subcortical networks, with the leading initial epileptogenic hubs in the cortical region, mainly frontal lobe. Overall the current neuromagnetic data in GGE shows the important role of earlier cortical involvement, mainly frontal and parietal regions, before triggering the rapid synchronization of the subcortical and cortical networks, which goes along with the *concept of cortical focus theory* (22, 23, 25). The hypothesis mentioned above is ascertained by the current literature listed above in Tables 1–3.

Moreover, all published data suggests that generalized epilepsy has increased focal epileptogenic hubs, i.e., uneven cortical excitability in mainly frontal or central or parietal regions depending on the types of the GGE, with rapid recruitment *via* cortico-thalamic oscillation to various topographic locations, rather than the diffuse involvement of the whole brain. With the availability of directed connectivity analysis, the presence of focal hyper-connectivity in the setting of the global network has been demonstrated. As described above, one of the particular challenges in the clinical setting is accurately categorizing epileptic patients into either focal epilepsy or generalized epilepsy as both have different treatment options in terms of ASM and non–pharmacological treatment (131). In some particular cases, it is very challenging to give an accurate diagnosis. In addition, one doesn't want to miss the epilepsy surgery opportunity window in focal epileptic patients with rich connectivity, especially in the pediatric population, as the patient is misclassified as generalized epilepsy. In contrast, one doesn't want to undergo expensive pre-surgical epilepsy workups in patients with generalized epilepsy. At present, there is no scientifically proven diagnostic biomarker available for these types of challenging cases, but there are some promising findings by analyzing the neuromagnetic data. As illustrated above, during the resting state, connectivity patterns are different between healthy control, focal epilepsy, and GGE. In GGE, there is a presence of disorganization in the default mode network (GGE, JME, and AE), frontoparietal network (AE), and sensorimotor network (JME) during the resting state. In contrast, in focal frontal lobe epilepsy, there is only focal hyperconnectivity in the frontal lobe. Thus, the difference between resting MEG connectivity analyses can be a promising diagnostic biomarker to differentiate between focal and generalized epilepsy. One of the well known challenges of network analysis is that there is no one method superior to the others, and thus lack of standardized methodologies will be perplexing for future research. More investigations with increased subjects are also warranted to compare GE resting connectivity with other types of focal epilepsy with high connectivity, such as posterior quadrant epilepsy, for possible diagnostic and prognostic biomarkers in epilepsy.

In the default mode network, basic network node regions are responsible for basic incoming and outgoing information, remains activated when an individual is not engaged in external tasks, whereas the default state is suspended if the individual concentrates on a task (154). Compared with controls, effective connectivity at the posterior cingulate and parietal cortex, which are part of the default mode network, is decreased in patients with CAE, suggesting PC/PCC might be crucial for consciousness (8). In addition, the reduced resting functional connectivity in PCC/pC is also reported in patients with attention-deficit disorder (130) and memory impairment. Thus, given the above finding, patients with CAE have a higher chance of attention deficit disorder (131). In JME, in addition to other networks, there is an altered resting-state sensorimotor network, and hence it may be a reason for the seizure-prone (motor) states in the JME. Thus among different GGE subtypes, there are different networks involved. In epilepsy, quality of life is dependent not only on seizure frequency but also on the presence of co-morbidities, such as learning disability, anxiety, and ADHD (131). Without a doubt, understanding the basic pathophysiology of GGE will enlighten the clinicians with more therapeutic targets to improve the quality of life in patients with GGE.

There are promising preliminary neuromagnetic data on the prognostic biomarkers for drug resistance in patients with the CAE. CAE patients with the presence of the ictal HFOs (250–1,000 Hz), localized to the medial prefrontal cortex, are associated with increased seizure frequency (115, 116, 118). Both ETX and LTG non-responders have increased pretreatment ictal local frontal connectivity and decreased anteroposterior /frontoparietal connectivity compared to non-responders (47, 128, 129). Thus, by exploring the pretreatment ictal HFO and resting-state connectivity of CAE patients, one may be able to predict whether the patient will be an ASM responder or non-responder. However, at this time, no causal assumption can be made between the ASM non-responsiveness and the ictal frontal and decreased anteroposterior connectivity due to the limited data. Further studies are needed to confirm the hypothesis using a large cohort prospective study with a longer follow-up duration.

More and more data suggest that the alterations in the connectivity of various networks in patients with GGE are more complex and maybe even more dynamic with various multi-directionality. As mentioned above, Tenney et al. (47, 48) combined MEG with fMRI, which improved the source localization over the sub-regions of the deep brain area, such as different parts of the basal ganglia, and subregions of thalamus could be explored as the different parts of the basal ganglia and thalamus has different connectivity and functionality. In addition, the same research group already presented cross-frequency coupling showing how dynamic changes occurred in the various network in the CAE at the preictal stage (47, 48). Although fMRI has better spatial resolution than MEG, it is still insufficient to accurately localize the neuromagnetic source to subnuclei of the thalamus (46). In patients with drug-resistant GGE, one currently available alternative treatment option after failing the multiple ASMs is neuromodulation. The treatment outcome of neurostimulators, mainly DBS, is highly dependent upon the locations of the electrodes placement, stimulation parameters, subtypes of generalized epilepsy, or even individual cortical-subcortical connectivity profile (20, 21, 155). Thus, further studies using multimodality analysis combining various advanced postprocessing neuromagnetic analysis and neuroimaging may enlighten the underlying pathophysiology of underlying network alteration in various ictal or interictal stages of the patients with various types of IGG in order to improve the treatment options in generalized epilepsy,

Since absence epilepsy is the most common GGE, has frequent seizures, and reduced movement artifact, most of the current literature on GSWDs has experimented on patients with AE, mainly CAE. It is unclear whether research findings for CAE can be generalized to all the various subtypes of generalized epilepsy. Given all the literature mentioned above, different subtypes of generalized epilepsy may have shared mechanisms or connectivity pathways, but this review clearly illustrates varied topographic cortical involvements in different generalized epilepsy based on their symptomatology. Hence, further studies are warranted to confirm this point of view.

Last but not least, another major limitation is how one can confirm the findings of the current non–invasive neuromagnetic data to support the concept of cortical focus theory, in which a highly connective cortical epileptogenic focus, most likely frontal hyperexcitability and parietal deactivation, triggering the rapidly generalized epileptic discharges involving intact corticothalamic or corticocortical networks. The finding has been confirmed in the animal model, with the cortical focus activation being found to be leading the thalamus activation by 500 ms (156). Although the ideal confirmation of the concept in humans should be analyzing intracranial invasive electrical activities from simultaneous cortical regions, covering bilateral multi-lobar regions, and various subcortical regions, subnuclei of bilateral thalami, it will be unethical and impractical to put multi-electrodes to cover every aspect of the thalamus and cortical regions. So far, a small study of intraoperative simultaneous invasive centromedian thalamic nuclei and scalp EEG recording had shown that generalized paroxysmal fast activity in patients with the Lennox-Gastaut syndrome appeared 75 ms later in thalamic activation when compared to the scalp frontal EEG activity, supporting a cortical driven process in generalized epilepsy (157). Another study investigated the interval relationship of the centromedian thalamus in relation to the cortical electrical activities in two patients with idiopathic generalized epilepsy (158). One of the two patients had bilateral independent discharges restricted only to the bilateral centromedian thalami, and the other had bilateral cortical discharges with the belated onset of leading thalamic discharges at the ictal onset (158). Thus, based on their symptomatology, the currently available data suggested there were different topographic cortical involvements in different subtypes of generalized epilepsy. Given the small sample size, no particular conclusion could be made. However, the findings from the CAE may likely be unable to generalize to all the subtypes of generalized epilepsy. Hence, further studies are warranted for the emerging development of responsive neurostimulation therapies for patients with generalized epilepsy.

In conclusion, current MEG literature challenges the concept of generalized epilepsy being fully generalized. Advances in recent MEG methodology contribute to the literature of idiopathic/genetic generalized epilepsy in terms of physiopathology, treatment and prognosis options, thus further blurring the boundary between focal and generalized epilepsy.

## Limitations

This review is limited because only three databases were searched by one reviewer (TA) and included only the published publication in English. All the posters publications were excluded. Thus, some of the remarkable pertinent studies might be missed in the literature review.

## Author Contributions

TA contributed to the conception, performed literature review, drafting the manuscript, revising the manuscript, and final approval of the version to be published. JT and AB contributed to the conception, revising the manuscript, and final approval of the version to be published. All authors contributed to the article and approved the submitted version.

## Conflict of Interest

The authors declare that the research was conducted in the absence of any commercial or financial relationships that could be construed as a potential conflict of interest.

## Publisher's Note

All claims expressed in this article are solely those of the authors and do not necessarily represent those of their affiliated organizations, or those of the publisher, the editors and the reviewers. Any product that may be evaluated in this article, or claim that may be made by its manufacturer, is not guaranteed or endorsed by the publisher.

## Abbreviations

GSWD: generalized spikes wave discharge
IEDs: Interictal epileptiform discharges
CAE: childhood absence epilepsy
JAE: juvenile absence epilepsy
JME: juvenile myoclonus epilepsy
GGE: genetic generalized epilepsy
TCS: tonic-clonic seizure
MEG: magnetoencephalography
EC: effective connectivity
EEG: electroencephalography
fMRI: functional magnetic resonance imaging
SAM: synthetic aperture magnetometry
LORETA: standardized low-resolution brain electromagnetic topography
sLORETA: standardized low-resolution brain electromagnetic topography
ms: milliseconds
LCMV: Linear constraint minimum variance
ASI: accumulated source imaging
DICS: Dynamic imaging of coherent sources
dMSI: Dynamic magnetic source imaging
dSPM: Dynamic statistical parameter mapping
ECD: equivalent dipole model
PLV: phase-locking value
MUSIC: multiple signal characteristic
wMNE: weighted minimum-norm estimation
pMEM: pairwise maximum entropy model
CFC: cross-frequency coupling
ms: milliseconds
s: seconds
m: months
y: years
F: female
M: male
ASM: antiseizure medication.
